# *FMR1*-dependent variability of ovarian aging patterns is already apparent in young oocyte donors

**DOI:** 10.1186/1477-7827-11-80

**Published:** 2013-08-16

**Authors:** Norbert Gleicher, Ann Kim, David H Barad, Aya Shohat-Tal, Emanuela Lazzaroni, Tamar Michaeli, Ho-Joon Lee, Vitaly A Kushnir, Andrea Weghofer

**Affiliations:** 1 Center for Human Reproduction, 10021, New York, NY, USA; 2 Foundation for Reproductive Medicine, 10021, New York, NY, USA; 3 Department of Gynecologic Endocrinology and Reproductive Medicine, Medical University Vienna, 1090, Vienna, Austria

**Keywords:** Fragile X gene, *FMR1* gene, Ovarian reserve, Anti-müllerian hormone (AMH), Oocyte donor

## Abstract

**Background:**

Hypothesizing that redundant functional ovarian reserve (FOR) at young ages may clinically obfuscate prematurely diminished FOR (PDFOR), we investigated in young oocyte donors genotypes and sub-genotypes of the *FMR1* gene, in prior studies associated with specific ovarian aging patterns, and determined whether they already at such young age were associated with variations in ovarian reserve (OR). We also investigated racial as well as *FMR1* associations with menarcheal age in these donors.

**Methods:**

In a cohort study we investigated 157 oocyte donor candidates and, based on the 95% CI of AMH, divided them into normal age-specific (AMH greater or equal to 2.1 ng/mL; n = 121) and PDFOR (AMH < 2.1 ng/mL; n = 36). We then assessed associations between numbers of trinucleotide repeat (CGGn) on the *FMR1* gene and FOR (based on anti-Müllerian hormone, AMH).

**Results:**

*FMR1* did not associate with AMH overall. Amongst 36 donors with PDFOR, 17 (42%) presented with at least one *low* (CGGn < 26 ) allele. Remaining donors with normal FOR presented with significantly more CGGn greater or equal to 26 (73.6% vs. 26.4%; P = 0.024) and higher AMH (P = 0.012). This finding was mostly the consequence of interaction between *FMR1* (CGGn < 26 vs. CGGn greater or equal to 26) and race (P = 0.013), with Asians most responsible (P = 0.009). Menarcheal age was in donors with normal FOR neither associated with race nor with *FMR1* status. In donors with PDFOR race was statistically associated with CGGn (P = 0.018), an association primarily based on significantly delayed age of menarche in African donors with CGGn < 26 in comparison to African donors with CGGn greater or equal to 26 (P = 0.019), and Caucasian (P = 0.017) and Asian donors (P = 0.025) with CGGn < 26.

**Conclusions:**

CGGn on *FMR1* already at young ages affects FOR, but is clinically apparent only in cases of PDFOR. Screening for *low FMR1* CGGn < 26 at young age, thus, appears predictive of later PDFOR.

## Background

The *FMR1* gene, located on the X chromosome, is widely tested in prenatal medicine in attempts to prevent the so-called fragile X syndrome, representing the one-generational expansion of a premutation-range CGG_n=55–200_ to a full mutation, characterized by a CGG_n>200_. Based on a newly defined normal range of CGG_n=26–34_, it has only recently in a number of publications been associated with ovarian aging. Different mutations of the gene appear associated with different rates of follicle depletion.

Lledo et al. recently, however, suggested that *FMR1* screening should not be considered in pre-assessing potential responses to ovarian stimulation [1], implying that different *FMR1* mutations do not correlate with variations in functional ovarian reserve (FOR). We, in contrast, previously reported specific ovarian *FMR1* genotypes and sub-genotypes, defined by normal range of CGG_n=26–34_ (median, CGG_n=30_), associated with distinct ovarian aging patterns [2,3]. These varying mutations of the *FMR1* gene have since also proven associated with risk towards autoimmunity [4] and, potentially, *BRCA1/BRCA2*-associated cancer risks [5]. Moreover, Hoffman et al. recently reported in a mouse analogue of human premutation range (CGG_n=55–200_) primary ovarian insufficiency (POI) that the model, in comparison to wild type mice, demonstrated clear anatomic evidence of premature ovarian aging (POA), including accelerated follicle loss [6].

Lledo and associates reached their conclusions, investigating oocyte yields in young oocyte donors [1]. Like them, we previously also had been unable to demonstrate differences in FOR in young oocyte donors [2]. We, therefore, to a degree based on their publication, hypothesized that young oocyte donors, likely, do not represent suitable subjects for such studies, as, at such young ages, even impeded FOR may still create enough redundancy to produce excellent oocyte yields [4].

Our earlier study involved, however, only 34 young women [2]. We, therefore, decided to revisit the question based on a larger patient sample, and a more sophisticated study protocol. We here, therefore, investigated a much larger egg donor population, stratified for normal FOR and PDFOR.

## Methods

This study investigated 157 consecutive oocyte donors, selected for our center’s oocyte donor pool. We investigated in this patient cohort whether their AMH levels at time of initial presentation varied in association with different *FMR1* mutations, adjusted for race. We, in addition, also investigated whether the donors’ menarcheal age was affected by either race and/or *FMR1* status.

Less than five percent of applicants are accepted into our center’s oocyte donor pool. Donor selection involves an initial screening step by questionnaire, followed by two rounds of face-to-face interview and a final medical testing round. Once candidates have passed this final testing round, they become eligible for matching with recipients. Once selected by a recipient, the donor undergoes a second testing round in accordance with guidelines issued by the United States Food and Drug Administration, the federal agency overseeing gamete donations in the United States. Only if this testing round is satisfactory is the donor considered “matched”.

In this study AMH and *FMR1* testing was performed on the day of the donor’s second face-to-face interview at the center. All patients at our center undergo routine *FMR1* testing to screen for one-generational expansion risk in offspring towards the fragile X syndrome. The test is neither performed nor used to predict reproductive performance of potential donors. Donor selection is, therefore, not influenced by what genotypes or sub-genotypes of *FMR1* a given donor candidate represents. Our center’s Institutional Review Board (IRB), however, based on prior published *FMR1* studies from our center, requires that donor candidates be informed if their *FMR1* mutations are believed to potentially denote risk towards PDFOR.

Ovarian *FMR1* genotypes and sub-genotypes, as previously described, are based on a normal CGG_n=26–34_ range [2,3]. This means that patients are considered to have normal (*norm*) genotypes if both alleles are in normal range. They are considered to be heterozygous (*het*) if one allele is outside, and homozygous (*hom*) if both alleles are outside of normal range. Depending on whether abnormal alleles are above or below normal range, *het* and *hom* genotypes are further sub-divided into sub-genotypes. *Het* genotypes are sub-divided into *het-norm/high* and *het-norm/low* and *hom* genotypes into *hom-high/high*, *hom-high/low* and *hom-low/low*. Every allele of CGG_n<26_ is, therefore, considered a *low* allele. *Norm* and *high* alleles in this manuscript are combined as CGG_n≥26_. *FMR1* and AMH assays were performed by routine commercial assays, as previously reported [2,3].

At time of acceptance into our center’s donor pool, the mean age of donors was 24.5 ± 3.2 years, their body mass index (BMI) was 21.3 ± 2.4 kg/m^2^ and their mean AMH was 4.2 ± 2.6 ng/mL (Table 1).

**Table 1:** Baseline characteristics of all oocyte donors

**Number of donor candidates**	**157**
Caucasian (%)	109 (69.4)
Asian (%)	26 (16.6)
African (%)	22 (14.0)
Age at presentation (years)	24.6 ± 3.2
Age at menarche (years)	12.9 ± 1.5
BMI (kg/m2)	21.3 ± 2.4
AMH (ng/mL)	4.2 ± 2.6
*FMR1* [n (%)]: norm	83 (52.9)
*het*	55 (35.0)
*het*–norm/high	16 (10.2)
*het*–norm/low	39 (24.8)
**hom**	19 (12.1)
hom-high/high	9 (5.7)
hom-high/low	5 (3.2)
hom-low/low	5 (3.2)

The lower 95% CI for all AMH values, at <2.1 ng/mL, was defined as diagnostic for PDFOR. Donors with AMH above this range were, therefore, considered to have normal FOR.

Because distribution patterns of *FMR1* genotypes and sub-genotypes vary between races [7,8], all data were adjusted for race. Racial assignments were made according to National Institutes of Health Criteria, with Hispanic donors assigned according to their racial self-definition.

Differences between continuous and categorical data were calculated using Kruskal-Wallis, Mann–Whitney U, two-way analysis of variance and Chi-Square tests, with P < 0.05 considered statistically significant. All post hoc procedures were performed using the Sidak test. Statistical analyses were undertaken using the Statistical Package for the Social Sciences 17.0 (SPSS, Chicago, IL, USA).

Oocyte donor candidates, like all other patients at our center, at time of initial consultation sign an informed consent, which allows for use of their medical record for research purposes, as long as the patient’s identity is protected and the medical record remains confidential. Both conditions were met for this study, and the study, therefore, qualified for expedited review by the center’s IRB. Like infertility patients, oocyte donor candidates also sign specific informed consents for genetic testing, including *FMR1* testing. Though, as already noted before, *FMR1* test results in our program are not utilized to select oocyte donors, our IRB mandated that donors be informed about the potential clinical significance if they demonstrated other than *norm* genotypes.

## Results

Table 1 summarizes the characteristics of all 157 oocyte donor candidates. As the table demonstrates over two-thirds were Caucasian; the remaining almost evenly split between women of Asian and African descent.

The *FMR1* genotype distribution was 83 *norm* (52.9%), 55 *het* (35.0%) and 19 *hom* (12.1%). *Het* were further sub-divided into 16 *het-norm/high* (10.2%) and 39 *het-norm/low* (24.8%), while *hom* was sub-divided into 9 *hom-high/high* (5.7%), 5 *hom-high/low* (3.2%) and 5 *hom-low/low* (3.2%). A total of 49 (31.2%) donors, therefore, demonstrated at least one *low* allele of CGG_n<26_, while 108 donors presented exclusively with CGG_n≥26_, representing *norm* (CGG_n=26–34_) or *high* (CGG_n>34_) alleles.

**Figure 1:**
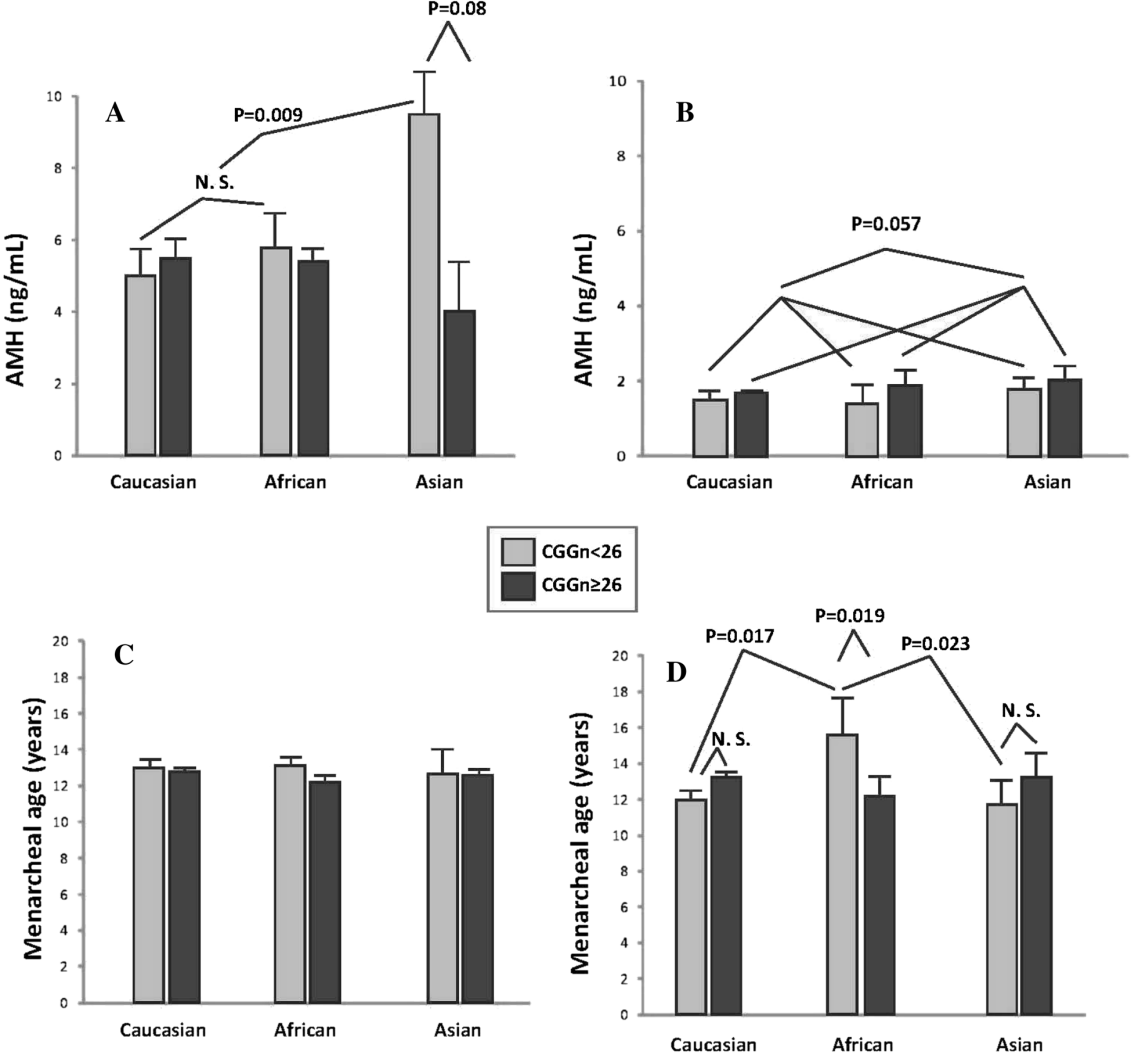
**AMH levels and menarcheal age in donors with normal and PDFOR. (A)** AMH levels, based on CGG_n_ on the *FMR1* gene in donors with normal FOR: AMH levels in the whole group differed significantly, based on *FMR1* (P = 0.012), with the difference primarily being due to Asian patients (see text). Independent of *FMR1*, the figure demonstrates similar AMH levels in Caucasian and African donors. Asian women with *low* (CGG_n<26_) allele, however, demonstrate significantly higher AMH (P = 0.009) than Caucasians and Africans combined, and Asian women with CGG_n=≥26_ (P = 0.006); **(B)** demonstrates AMH levels in donors with PDFOR: here, all three races demonstrate lower AMH with *low FMR1* allele, the difference almost reaching significance (P = 0.057); **(C)** demonstrates that with normal FOR age of menarche did not differ based on either race or *FMR1* status; **(D)** in contrast, demonstrates with PDFOR there was significant interaction between CGG_n_ and race (P = 0.018), with African donors reaching menarche at later age than Caucasians (P = 0.017) and Asians (P = 0.025), and amongst African donors those with CGG_n<26_ reaching menarche later than those with CGG_n≥26_ (P = 0.019).

Figures 1A, B present FOR data, based on AMH measurements, with A representing donors with normal FOR and B representing donors with PDFOR. Differences are obvious, with normal FOR donors demonstrating uniformly mean AMH levels above 4 ng/mL, while donors with PDFOR uniformly demonstrate mean AMH below 2 ng/mL.

When the 36 donors with PDFOR (AMH < 2.1 ng/mL) were investigated separately, differences started coming into focus: Among 36 donors with PDFOR, 17 (47.2%) presented with at least one *low FMR1* allele (CGG_n<26_) and 19 (52.8%) with none. The remaining 121 donors with normal FOR (AMH ≥ 2.1 ng/mL) were significantly more likely to have a CGG_n≥26_ allele (73.6% vs. 26.4%; P = 0.024).

Normal FOR donors with a *low* (CGG_n<26_) allele still demonstrated higher mean AMH levels than donors with CGG_n≥26_ (P = 0.012). As Figure 1A demonstrates, amongst Caucasian and African donors AMH levels were similar and did not differ significantly based on CGG_n_. Asian donors, however, demonstrated, in comparison, significantly higher AMH in association with presence of at least one *low* (CGG_n<26_ ) allele (P = 0.009). They, therefore, are likely primarily responsible for above described statistical association with CGG_n_ for the whole group of donors with PDFOR.

These findings need to be contrasted to AMH levels in donors with PDFOR, where, independent of race, all donors presented with much lower AMH overall, but with higher AMH levels in absence of a *low* allele (Figure 1B) in each race, establishing an almost significant trend (P = 0.057) in reversal to observation in donors with normal FOR. Figure 1B further demonstrates that Caucasians and African donors once again demonstrated similar AMH levels, while Asian donors, again, demonstrated highest AMH levels, here, however, independent of CGG_n_.

When investigating menarcheal age, neither race nor *FMR1* status mattered in donors with normal FOR (Figure 1C). Donors with PDFOR, however, demonstrated significant differences by demonstrating significant interaction between *FMR1* CGG_n_ and race (P = 0.018), primarily based on significantly later menarche in African women with *low* CGG_n<26_. African donors with *low* CGG_<26_ demonstrated significantly later menarche than African donors with CGG_n≥26_ (P = 0.019), and than *low FMR1* Caucasian (P = 0.017) and Asian (P = 0.025) donors (Figure 1D).

## Discussion

A so-called premutation-range genotype (CGG_n=55–200_) of the *FMR1* gene has for decades been associated with highly increased risk towards primary ovarian insufficiency (POI), also called premature ovarian failure (POF) [6,9]. Specific ovarian functions of the *FMR1* gene have, however, only in recent years been described in association with newly described *FMR1* mutations, which were established based on a normal range of CGG_n=26–34_[2,3].

We started to suspect a specific ovarian function of *FMR1* after noting a large distribution peak at CGG_n=31–32_ in a quite dated publication by Fu and associates [10], suggesting a potentially new normal CGG_n_ distribution range for a new function of the gene. In assessing CGG_n_ in various populations, we then demonstrated that, independent of racial background, a normal “ovarian” CGG triple nucleotide range, indeed, appeared at CGG_n=26–34,_ close to, and including the distribution peak reported by Fu et al. [2,11]. Defining such a normal range we then, based on CGG_n_, can classify her as *norm*, *het* and *hom*[2]. *Het* and *hom* genotypes can be further sub-divided, based on whether abnormal alleles have CGG_n_ above (*high*) or below (*low*) normal range [3].

*FMR1* genotypes and sub-genotypes have since been associated with different ovarian aging patterns [2,3], different pregnancy rates in association with in vitro fertilization (IVF) [3] but also with risk towards autoimmunity [3,8] and, likely, *BRCA1/2* mutation-associated cancer risks [5], suggesting, yet additional, previously unknown biological functions for the *FMR1* gene.

The most interesting evolving mutation of *FMR1*, so far, has been the *het-norm/low* sub-genotype, characterized by one allele in *norm* range and one at CGG_n<26_. This *low* sub-genotype has been associated with a polycystic ovary-like ovarian phenotype, which, because of rapid recruitment, and therefore depletion of FOR, quickly deteriorates into a phenotype characterized by premature diminished FOR [3]. It is also the genotype associated with lowest IVF pregnancy rates [3], autoimmunity [3,5] and, likely, *BRCA1/2*-associated cancer risks [5]. Interestingly, its “sibling” sub-genotype, *het-norm/high*, is associated with highest FOR at very advanced female ages [12], and is practically protective of autoimmune [3] and *BRCA1/2*-associated cancer risks [5].

Here presented study for the first time offered the opportunity to examine the effects of the *FMR1* gene on the ovarian aging process in a very young, and apparently normal cohort of females: young egg donors. For the complete study cohort, CGG_n_ of the *FMR1* gene did not affect OR. *FMR1* effects, however, did become apparent in donors who already at such young age (mean 24.6 years) suffered from PDFOR.

The study included 36 such donors, 17 (47.2%) with a *low* allele and 19 (52.8%) without. Donors with PDFOR were significantly less likely to present with CGG_n≥26_ than donors with normal FOR (P = 0.024). In young women with absolutely normal FOR, redundancy of extra ovarian reserve, therefore, indeed, appears substantial enough to prevent *FMR1*-driven differences, caused by *low FMR1* mutations, to become clinically apparent. We suspected so much in a response to the recent publication by Lledo et al. [1].

Higher AMH values, as observed in African and Asian donors with normal FOR and *low FMR1* CGG_n<26_ than in donors with CGG_n≥26_ (P = 0.012, Figure 1A), may at first glance surprise since higher AMH denotes better FOR, resulting in better quantitative and qualitative IVF outcomes [13]. Since these high AMH values appear primarily the consequence of observations in Asian women (Figure 1A), they, however, have to be viewed with caution because young Asian women are not typically known to present with high FOR (7,8). Moreover, though *low FMR1* mutations of the *het-norm/low* sub-genotype have been associated with the poorest IVF outcomes amongst all *FMR1* genotypes [3], this observation, too, has to be understood within a proper context.

The *het-norm/low* sub-genotype of the *FMR1* gene is at young ages characterized by a PCOS-like ovarian phenotype. PCOS, in turn, is associated with high FOR and, therefore, high AMH levels. Young women with *low FMR1* mutations, while still during the PCOS-like phase of their ovarian development, therefore, are expected to demonstrate high AMH values. Considering small numbers of Asian and African donors in this study, here observed high AMH levels, especially amongst Asian donors, have to be viewed with caution. Reported studies actually suggest a preponderance of *low FMR1* alleles in African rather than Asian women [7,8].

This PCOS-like ovarian phenotype in *low FMR1* mutation carriers is, because of relatively rapid recruitment, also characterized by early follicle depletion, by mid-age transforming many of these women into patients with PDFOR [3]. Young oocyte donors with *low FMR1* alleles and high FOR, therefore, may reflect ovarian phenotypes pre-depletion.

This interpretation of here reported data is also supported by the previously noted distribution of *FMR1* mutations amongst donors: Those who already had entered PDFOR (n = 36), in almost half of cases (17/36; 47.2%) demonstrated *low FMR1* alleles, while those with normal FOR exhibited CGG_n<26_ in only 32/121 (26.5%; P = 0.018).

Inversion of AMH data, as demonstrated in Figures 1A (normal FOR; overall higher AMH with CGG_n<26_) and 1B (PDFOR; higher AMH with CGG_n≥26_), therefore, may or may not be race-dependent. Data, however, do appear to suggest that the *FMR1* gene, indeed, does affect FOR already at the young ages, though such effects are in many young women not yet detectable by AMH assessments at such early stages. Here presented data, therefore, are in agreement with previously published *FMR1* data in older infertile patients, which suggest that a *low* allele is prognostically predictive of PRFOR and poorer pregnancy chances [3].

These data, however, in addition for the first time in exclusively young, and apparently healthy females demonstrate that a *low FMR1* CGG_n<26_ allele appears predictive of PDFOR. While earlier cross-sectional studies from our center in infertile patient populations were suggestive of such an interpretation, here presented data offer convincing evidence in a very young, and seemingly fertile, highly selected egg donor population.

Screening for *low FMR1* mutations in young women, therefore, may offer the opportunity to detect genetically predisposed women towards PDFOR already at very young ages. Confirmation of risk by detection of a low *FMR1* allele then would allow for longitudinal FOR assessments with AMH and FSH to either confirm or refute a diagnosis of PDFOR.

This study also demonstrates that, even in the early 20s, some young women with *low FMR1* mutations, and therefore at risk for PDFOR, still have so much overabundant FOR that AMH measurements will not yet allow for a diagnosis. Such women, therefore, will have to be followed longitudinally with serial AMH and FSH measurements. Confirmation of PDFOR at much younger age than when currently PDFOR diagnoses are reached, will allow affected women to plan their reproductive life accordingly and/or consider fertility preservation through social oocyte cryopreservation with much better success and at lower cost than with later diagnosis.

The importance of *low FMR1* mutations for the ovarian aging process is further emphasized by here reported menarcheal age data. While the total donor population again does not reveal any race- or *FMR1*-based differences (Figure 1C), donors with PDFOR do demonstrate significant differences based on race and *FMR1* mutation (Figure 1D). Here, significant interaction between CGG_n_ and race became apparent (P = 0.018), with African donors entering menarche at older age than either Caucasians or Asians.

That African donors behave so differently from Asian and Caucasian donors has, once again, to be viewed with caution and considered preliminary, in view of the relatively small size of the African donor pool in this study. Studying much older infertile women, we recently reported that, adjusted for age and race, diminished functional ovarian reserve (DFOR) in infertile women was statistically highly associated with younger age at menarche [13]. Since among all genotypes and sub-genotypes *low FMR1* mutations appear most closely associated with DFOR, one would expect to find younger menarcheal age amongst donors with CGG_n<26_ than amongst donor with CGG_n≥26_. That Asians demonstrate such a trend and Caucasians to a lesser extent but that Africans demonstrate to a significant degree exactly the opposite (Figure 1D) may suggest racial differences as to how the *FMR1* gene affects ovarian aging. This study, however, does not allow for further comments on the subject.

This study’s major weakness is the small size (n = 36) of donors with PDFOR, as they ended up representing the primary study population, demonstrating significant positive associations. Considering the small size of this cohort, the robustness of here reported statistical findings is actually rather surprising. Our findings, however, do require confirmation in larger young patient populations with PDFOR before here proposed conclusions can be considered confirmed.

Maybe the most surprising finding of this study was, however, the undisputed observation that 36/157 (22.9%) of carefully selected young oocyte donors, based on 95% CI for AMH, were found with great likelihood to suffer from PDFOR. This observation demonstrates the limitations of currently used oocyte donor selection methods. As noted under Materials and Methods, our center, based on instructions from our center’s IRB, currently does not utilize *FMR1* genotypes and sub-genotypes in egg donor selection. Here presented data suggest that *FMR1*-testing may, however, indeed, have a place in the egg donor selection process.

Finally, this study, once again, points to the importance of racial differences in reproductive medicine. We on repeated occasions have made this point in recent publications [4,11], stressing the importance of racial adjustments in studies of reproductive outcomes.

## Conclusions

CGGn on *FMR1* already at young ages affects FOR, but is clinically apparent only in cases of PDFOR. Screening for *low FMR1* CGG_n<26_ at young age, thus, appears predictive of later PDFOR.

## Competing interests

N.G, A.W. and D.H.B. have in the past received research support, speakers’ honoraria and travel funds from various pharmaceutical and medical device companies, none, however, related to the subject of this paper. N.G. and D.H.B. are listed as co-inventors of two awarded U.S. patents, claiming therapeutic benefits for DHEA, and potentially other androgens, in women with DOR. Both authors have other pending patent applications, regarding DHEA, and other androgens, and the FMR1 gene’s effects on ovaries. N.G. owns shares in Fertility Nutraceuticals, LLC, a company that offers a DHEA product. N.G. and D.H.B. are receiving patent royalties from this company. N.G. is also the owner of The CHR, where this research was conducted. Other authors have no competing interests to declare.

## References

[B1] Lledo B Guerrero J Ortiz JA Morales R Ten J Llacer J Gimenez J Bernabeu R Intermediate and normal sized CGG repeats on the FMR1 gene does not negatively affect donor ovarian response Hum Reprod 2012 27 609 614 10.1093/humrep/der415 22157911

[B2] Gleicher N Weghofer A Barad DH Ovarian reserve determinations suggest new function of FMR1 (fragile X gene) in regulating ovarian ageing Reprod Biomed Online 2010 20 768 755 10.1016/j.rbmo.2010.02.020 20378415

[B3] Gleicher N Weghofer A Lee IH Barad DH FMR1 genotype with autoimmune-associated polycystic ovary-like phenotype and decreased pregnancy chances PLoS One 2010 5 315303 10.1371/journal.pone.0015303 PMC3002956 21179569

[B4] Gleicher N Weghofer A Barad DH Intermediate and normal sized CGG repeats on the FMR1 gene does not negatively affect donor ovarian response Hum Reprod 2012 27 7 2241 2241 author reply 2242–2243 22617125 10.1093/humrep/des161

[B5] Weghofer A Tea M-K Barad DH Kim A Singer CF Wagner K Gleicher N RCA1/2 mutations appear embryo lethal in humans, unless rescued by low (GCCn < 26) FMR1 sub-genotypes PLoS One 2012 in press 10.1371/journal.pone.0044753 PMC3440327 22984553

[B6] Hoffman G Le WW Entezam A Otsuka N Tong Z-B Nelson L Flaws JA McDonald JH Jafar S Usdin K Ovarian abnormalities in a mouse model of Fragile X Primary Insufficiency J Histochem Cytochem 2012 60 439 10.1369/0022155412441002 22470123 PMC3393073

[B7] Gleicher N Kim A Weghofer A Barad DH Differences in ovarian aging patterns between races are likely related to ovarian genotypes and sub-genotypes of the FMR1 gene Reprod Biol Endocrinol 2012 10 77 10.1186/1477-7827-10-77 22963248 PMC3495196

[B8] Gleicher N Weghofer A Lee IH Barad DH Association of FMR1 genotypes with in vitro fertilization (IVF) outcomes based on ethnicity/race PLoS One 2011 6 e18781 10.1371/journal.pone.0018781 21526209 PMC3078144

[B9] Wittenberger MD Hagerman RJ Sherman SL McConkie-Rosell A Welt CK Rebar RW Corrigan EC Simpson JL Nelson LM The FMR1 premutation and reproduction Fertil Steril 2007 87 456 465 10.1016/j.fertnstert.2006.09.004 17074338

[B10] Fu YJ Kuhl DP Pizzuti A Pieretti M Sutcliffe JS Richards S Verkerk AJ Holden JJ Fenwick RG Jr Warren ST Variation of the CGG repeat at the fragile X site results in genetic instability: resolution of the Sherman paradox Cell 1991 20 67 1074 1058 10.1016/0092-8674(91)90283-5 1760838

[B11] Gleicher N Weghofer A Oktay K Barad DH Effects of race/ethnicity on triple CGG counts on the FMR1 (fragile X) gene in infertile women Reprod Biomed Online 2010 20 485 491 10.1016/j.rbmo.2009.12.017 20149747

[B12] Gleicher N Weghofer A Kim A Barad DH The impact in older women of ovarian FMR1 genotypes and sub-genotypes on ovarian reserve PLoS One 2012 7 e33638 10.1371/journal.pone.0033638 22438971 PMC3306274

[B13] Weghofer A Kim A Barad DH Gleicher N Age at menarche: a predictor of diminished ovarian function? Fertil Steril 2013 [Epub ahead of print] 10.1016/j.fertnstert.2013.05.042 23809497

